# Artificial fast-adapting mechanoreceptor based on carbon nanotube percolating network

**DOI:** 10.1038/s41598-021-04483-2

**Published:** 2022-03-09

**Authors:** Cyril Bounakoff, Vincent Hayward, Jonathan Genest, François Michaud, Jacques Beauvais

**Affiliations:** 1 Department of Electrical Engineering and Computer Engineering, Interdisciplinary Institute for Technological Innovation (3IT), Université de Sherbrooke, Sherbrooke, QC Canada; 2 Sorbonne Université, Institut des Systèmes Intelligents et de Robotique, 75005 Paris, France; 3 School of Electrical Engineering and Computer Science, University of Ottawa, Ottawa, ON Canada

**Keywords:** Electrical and electronic engineering, Sensors, Biomimetic synthesis

## Abstract

Most biological sensors preferentially encode changes in a stimulus rather than the steady components. However, intrinsically phasic artificial mechanoreceptors have not yet been described. We constructed a phasic mechanoreceptor by encapsulating carbon nanotube film in a viscoelastic matrix supported by a rigid substrate. When stimulated by a spherical indenter the sensor response resembled the response of fast-adapting mammalian mechanoreceptors. We modelled these sensors from the properties of percolating conductive networks combined with nonlinear contact mechanics and discussed the implications of this finding.

## Introduction

Endowing robots with the dexterity and the agility of humans, primates, and other animals depends on providing them with equivalent sensing capabilities. In the natural world, prehensile and discriminative touch depend on phasic skin mechanoreceptors found in the glabrous skin of humans and animals. These mechanoreceptors adapt rapidly over time scales of the order of 100 ms^1–4^. Mechanoreceptors with tonic responses adapt with much greater time scales^5^.

In general, sensing is said to be phasic when only changes in a specific property of a stimulus elicit sensory responses. Phasic sensing is ubiquitous, not only in touch, but across all biological senses. Phasic sensory neurones are found in vision^6^, audition^7,8^, proprioception^9^, olfaction^10^, thermoception^11^, and in the vestibular system^12^.

The hairy skin, which covers much of the body except the volar regions of hands and feet, is noted for an absence of fast-adapting phasic receptors^13–15^. This absence correlates with the fact that hairy skin is normally not engaged in prehension or surface discrimination. A system of phasic sensory neurones, also innervating the human hairy skin, projects to the brain via slow-conducting nerve fibres. The phasic properties of these neurones have very long time scales (several seconds) and are thought to be contributing to limbic functions^16^.

Fast-adapting phasic mechanoreceptors are found in great densities in the extremities of mammals with soft pads. In the human fingertip, the density of phasic receptors is five times greater than that of tonic receptors^17^. A hallmark of phasic sensory neurones is to respond preferentially at the onset and at the termination of a stimulus by contrast with tonic sensory neurones that respond throughout the imposition of a stimulus^18–20^.

Receptors that exhibit phasic responses are termed adaptive because adaptation relates to a modification of the encoding of a stimulus according to its statistics. Adaptation comes with the fundamental advantage of optimising the use of physiological resources in an organism^21^. Simply said, changes in sensory stimuli are worthier to be encoded than the steady components.

Recent surveys indicate that the physics commonly harnessed to realise artificial tactile sensors include: piezoresistivity, capacitance, piezoelectricity, light transmission and reflection^22–27^. Piezoresistive sensors alter an electric current according to the strain to which a conductive element is exposed. Capacitive sensors relate the distance between two electrodes to a voltage or a frequency signal. Piezoelectric sensors displace charges according to the dimensional changes of a domain. Optical sensors grade a flow of photons according to the displacement of an element. All these sensors are intrinsically tonic because the signal is related to deflection. In robotics, the notion of phasic sensing is often associated to temporal high-pass filtering properties of individual mechano-sensing units^28–33^. Piezoelectric sensors in particular acquire high-pass characteristics when connected to low electrical impedances. The dynamic properties of these sensors are obtained by the readout circuitry, not by the transduction process.

From a biomimetic perspective, the availability of artificial sensors with intrinsic phasic properties is highly desirable according to the parsimonious principle of “morphological computation” where the physics of the embodiment of an organism perform functions that are relevant to perceptual or motor behaviour^34,35^. We describe here a mechano-sensing cell that exhibits an intrinsic phasic response without resorting to electronic circuitry. This attribute ties the delivered signal to changes in deflection rather than to deflection per se. These mechano-sensing cells, manufactured by embedding multi-wall carbon nanotubes (MWCNT) in a compliant polymer matrix, are soft, miniaturisable, printable, and compatible with large-scale production processes^24^.

## Results

**Figure 1:**
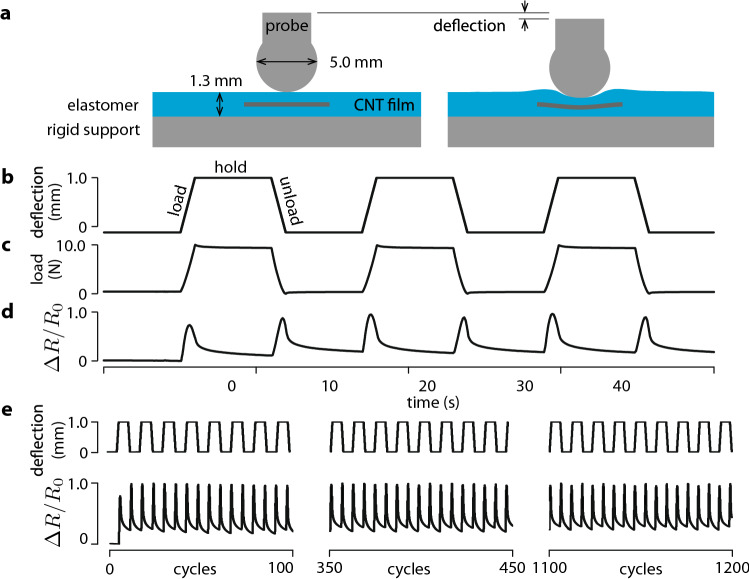
Sensor cell test by a spherical probe. (**a**) Sensor structure. (**b**) Testing sequence. (**c**) Resulting load. (**d**) Relative change of resistance $\Delta R = (R - R_0)/R_0$. (**e**) Repeated tests and responses.

Compliant tactile sensing cells were manufactured by embedding MWCNT films in a Polydimethylsiloxane (PDMS) matrix. The films formed a conductive percolation network that acted as a piezoresistive sensor with a resting resistance of $\simeq$ ${30}\, k \Omega$. The films were 12.5 $\times$ 2.5 $\times$ 0.5 mm strips embedded in a 1.3 mm-thick elastic matrix at a distance of 0.65 mm from the upper surface. The elastic layer was set on a rigid aluminum support, as in Fig. 1a. Several sensing cells were tested using a universal testing machine (“Methods”) that imposed sequences of indentations by a 5 mm spherical probe with a loading phase, a hold phase, and an unloading phase, as in Fig. 1b. In a given specimen, indentations produced the load variations shown in Fig. 1c and the relative variation of resistance of the film shown in Fig. 1d. Cells tested through thousands of loading cycles did not exhibit signs of damage, as shown in Fig. 1e. We tested ten samples of the same structure and same fabrication process. The maximal changes of resistance during the loading phases had a standard deviation of 2.3%, showing good precision across samples. The accuracy of the samples remained under 9.5% over 1500 cycles.

**Figure 2:**
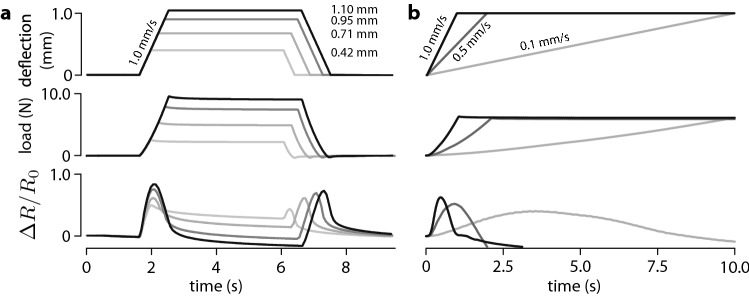
Sensor response. (**a**) Dependence of the response on deflection magnitude. Lines with grey levels indicate different deflections. (**b**) Dependence of the response on deflection rate. Lines with grey levels indicate different rates.

Surprisingly, the measurements indicated that relative change of resistance was the greatest during the loading and the unloading phases and remained low during the holding phase. In other words, the sensor responded mostly to changes in normal indentation. In addition, during steady loading, the response exhibited slow relaxation. Thus, the sensor cell had a response bearing an intriguing similarity with that of rapidly adaptive sensor cells found in mammalian skin^19^. A second experiment where indentation was varied for a same speed of indentation and where the speed of indentation was varied to reach the same indentation gave the results shown in Fig. 2a,b, respectively. The change of resistance during the loading and unloading phases was clearly a function both of the final magnitude of the indentation and of the rate of indentation, prompting us to investigate the mechanisms responsible for this surprising behaviour.

## Mechanism

### Empirical model

**Figure 3:**
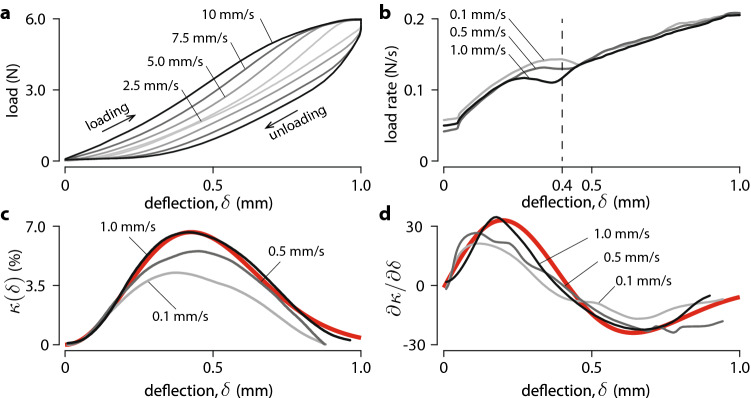
Large displacement analysis. (**a**) Rate dependent hysteresis. (**b**) Non-monotonic relationship between load-rate and deflection. (**c**) Relationship between the variation of resistance and deflection. The red line shows the prediction of the model (1). (**d**) Relationship between the rate of change of resistance and the deflection. The red line shows the prediction of the model (2).

Measurements showed nonlinear stiffening typical of Hertzian contact mechanics and rate-dependent hysteresis loops that were attributed to the viscoelasticity of the matrix as shown in Fig. 3a. A three-parameter generalised Maxwell model described this behaviour (see Supplementary Text S1). The load rate as a function of deflection, Fig. 3b, suggests that a change of deformation regime took place at around 0.4 mm of indentation.

Let $\kappa (\delta )$ relate the deflection, $\delta$, to the relative change in electrical resistance, $\kappa =\Delta R/ R_0$. Figure 3b,c plots this map for different indentation rates (same data as in Fig. 2b). The dependency on deflection is well captured by a smooth approximation (red line in Fig. 3c) expressed by,

1 $$\begin{aligned} \kappa (\delta ) \approx \delta ^2\,g(\delta ),\ \text {where}\quad g(\delta ) = a e^{-\frac{\delta ^2}{2\sigma ^2}}, \end{aligned}$$

where *a* is a scaling factor and $\sigma$ describes the spread of the response. The modelled response (1) did not depend on whether the sensor was loaded or unloaded. The rate of change of $\kappa$ with respect to $\delta$ (red line in Fig. 3d) is,

2 $$\begin{aligned} \frac{\mathrm {d}\kappa }{\mathrm {d}\delta } \approx \frac{\mathrm {d}[\delta ^2 g(\delta )]}{\mathrm {d}\delta } =\left( 2\delta - \frac{\delta ^3}{\sigma }\right) g(\delta ). \end{aligned}$$

Since $\delta$ is a function of time,

3 $$\begin{aligned} \frac{\mathrm {d}\kappa }{\mathrm {d}t} \approx {\dot{\delta }}\left( 2\delta - \frac{\delta ^3}{\sigma }\right) g(\delta ). \end{aligned}$$

Equation (3) captures the sensitivity of the sensor cell to the rate of indentation as it could be observed in Fig. 2b. The sensor response was quicker for higher indentation rates. Likewise, (3) predicts that for a same indentation rate, the response is higher for greater deflections, which can also be observed in Fig. 2a.

### Physical mechanism

The response captured by (1) must have been grounded in the physics of the sensor. The simplest model could follow from standard piezoresistivity (see Supplementary Text S2) which describes the relative variation of resistance of an elongated element. The model combines the intrinsic variation of specific resistance of the material, $\Delta \rho /\rho$, owed to the strain, $\varepsilon$, to which the element is exposed and the effect of dimensional changes represented by axial and transverse strains, $\varepsilon _{\mathrm{a}}$ and $\varepsilon _{\mathrm{t}}$, respectively. If $\nu$ is the Poisson ratio of the material, then,

4 $$\begin{aligned} \kappa (\varepsilon ) = \frac{\Delta \rho }{\rho }(\varepsilon ) + \varepsilon _{\mathrm{a}} (1 + 2\nu ) = \frac{\mathrm {d}\rho }{\rho }(\varepsilon ) - \varepsilon _{\mathrm{t}} \bigg (\frac{1}{\nu } + 2\bigg ). \end{aligned}$$

Thus, standard piezoresistivity model (4) cannot explain the reversal of the sign of the change of resistance empirically observed in Fig. 3c,d.

A more detailed model could appeal to the properties of percolation networks. Their behaviour is modelled by evaluating the average length and number of junctions established in an heterogenous conductor–insulator mixture^36^. When the number of junctions is assumed to be constant, the relative change of resistance takes the form (see Supplementary Text S3),

5 $$\begin{aligned} \kappa = e^{\gamma s_0 \varepsilon _{\mathrm{a}}} - 1 = e^{-2\gamma s_0 \varepsilon _{\mathrm{t}}} - 1, \end{aligned}$$

where $\gamma$ is a constant involving the mass and charge of an electron, Planck’s constant, and the potential barrier between nanotubes, and where $s_0$ is the effective distance between nanotubes in an undeformed film. An increase of resistance is attributed to an increase of the average distance between nanotubes that exponentially reduces the probability of electrons to tunnel through junctions. Slabs of CNT films were shown to verify this model^37,38^. Nevertheless, the nonlinear relationship expressed by (5) is still unable to explain the observed sign reversal of the change of resistance.

**Figure 4:**
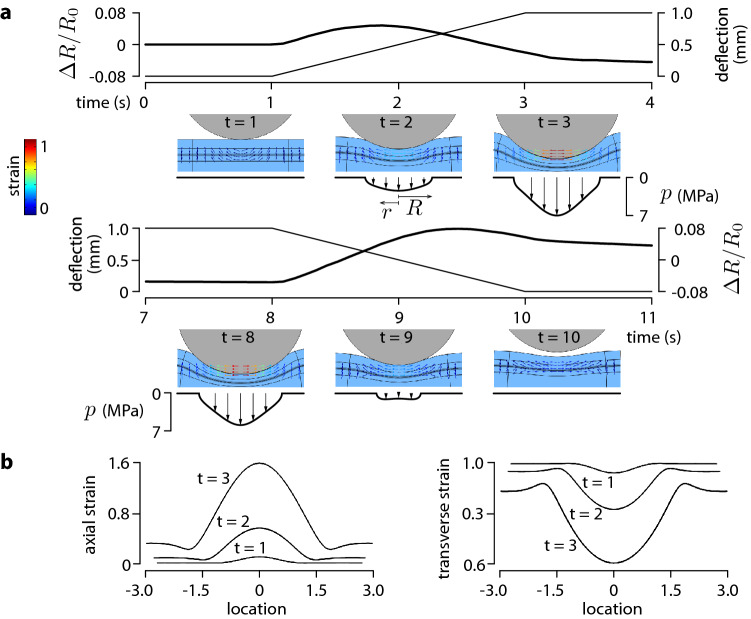
Finite element simulation. (**a**) The top sequence shows the result of the simulation of the progressive indentation by a cylindrical probe in the sensor cell. Arrows show direction and magnitude of principal strain. Beyond 0.4 mm of deflection, the behaviour departed from a Hertzian model as indicated by the pressure profile. The bottom sequence shows the unloading phase after a five-second plateau at fixed indentation. (**b**) Axial and transverse strain profiles along the MWCNT film during the loading phase.

To elucidate the mechanism behind the observed reversal of resistance, we set up a finite element simulation (2D planar case, COMSOL Multiphysics, Stockholm, Sweden) shown in Fig. 4a since there is no known closed-form solution to this contact problem^39,40^. The simulation suggested that the spherical probe deformed the sensitive film in a manner that initially resembled a Hertzian contact problem. However, the elastic layer mounted on a rigid support caused an infinite half-space approximation to no longer be applicable in the later stages of the indentation. The principal strains grew increasingly fast and a larger portion of film was recruited when the probe approached the support. This was confirmed by plotting the surface pressure profile, *p*(*r*), which at 0.7 mm of indentation departed significantly from the Hertz model, $p(r)\propto \sqrt{1-r^2/R^2}$. Figure 4b shows the axial and transverse strains along the length of the piezoresistive film during indentation. They clearly show that deformation rapidly transitions to a large strain regime over an increasingly larger region owing to the effect of the rigid support. The observed non-monotonic response can now be explained as follows.

Kang et al. subjected SWCNT nano-composite films to large tensile and compressive loads and found the relative resistance to increase with axial strain and decrease with compressive loads^37^. Their analysis suggested that changes in the tunnelling resistance were combined with an alteration of the band-gap in the junctions. Thus, the contact mechanics induced in an elastic film trapped between an indenter and a rigid support may have provoked a switch in the material state from a dominantly axial deformation that increased resistance to transverse deformation that decreased resistivity.

**Figure 5:**
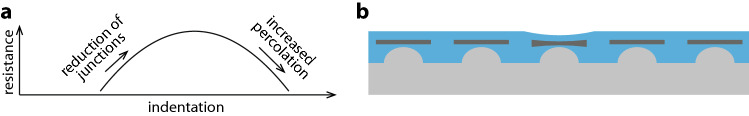
Qualitative model. (**a**) The interplay of two piezoresistive phenomena explains the sensitivity curve reversal. (**b**) Option for a practical e-Skin implementation.

The model (1) describes the empirical response of the sensor cell by the combination of these two effects, Fig. 5a. The viscoelasticity of the polymer matrix caused material relaxation under explaining the slow response at constant indentation and the asymmetry between the loading an unloading phases in the empirical measurement and in the simulation.

## Discussion

### A specific nonlinearity confers a phasic character

The complete response to indentation could be explained by a nonlinear model formed by the product of a Gaussian function, $g(\delta )$, with a parabola, $\delta ^2$, as in model (1). The dynamic response was then the product of the temporal rate of indentation with the rate of change of the response to indentation as in (3). The crucial point is the change of sign of the response’s rate of change, which, through mechanical nonlinear filtering, gave the sensor a phasic character. It only responded to changes in indentation. This property could also be captured by a Gaussian wavelet centred around a specific value of the indentation, $\delta _{\mathrm{r}}$, where the change of resistance as a function of indentation changes sign ($\delta _{\mathrm{r}}\approx {0.4}\, \hbox {mm}$ for the sample we tested). The derivative of the response with respect to indentation would then be given by a Gaussian multiplied by the first Hermite polynomial, $-\sigma ^{-2}(\delta -\delta _{\mathrm{r}})\,g(\delta -\delta _{\mathrm{r}})$. Rational expressions, splines, and others options are also available to represent the empirical results, $a\delta ^2/(b+\delta ^6)$, for example.

### Connection to biological sensors

Knibestöl^18^ found a diversity of responses to indentation rate in fast-adapting mechanoreceptors, with thresholds distributed over a hundred-fold span. He could developed a three-parameter sigmoidal-log model that accounted for the individual responses of twenty units. Iggo and Ogawa^19^ when attempting to disentangle the response to magnitude of indentation from rate of indentation, found a significant relationship between the rate of discharge and the rate of indentation and a weak relationship with magnitude. Burgess et al.^20^ also found a relationship between indentation rate and receptor response. Hayward et al.^41^ investigated the dependency of the neuronal response on the temporal-rate of mechanical stimuli in second-order cells. In mammals, second-order cells receive mono-synaptic inputs from a large number of primary afferents which, statistically, are mostly fast-adapting. Certain types of stimuli elicited responses that were strongly dependent on stimulus variations due to movement and therefore depend on phasic sensing. It may be speculated that the biological mechanoreceptors gain phasic properties through static nonlinearities of the type of model (1). Models of molecular mechanisms that produce specific phasic responses in mechanoreceptors are as of yet largely unknown^42–44^.

### Derivative designs

The sensory response described earlier can vary according to numerous design factors including the geometry of the surface impinging on the MWCNT film, the thickness of the film, the concentration of MWCNT, the material properties of the elastomer matrix in which they are embedded, the geometry and the material properties of the viscoelastic medium in which the film is embedded. The design space of sensing units based on similar principles is large.

Practical realisations could differ from the sensing unit studied in our experiments. For example, an inverted design with protuberances on the rigid support surface to obtain the desired nonlinear characteristics, as in Fig. 5b, would have advantages for use as an artificial skin.

### Relationship with event-based vision

In computer vision, “event-based cameras”^45^ that encode the change of brightness per pixel, and which are also bio-inspired sensors, share many characteristics with the sensing principle we have described. These characteristics include high temporal resolution, data transmission reduction, and the ability to serve as front-end to learning-based techniques such as spiking neural networks^46^.

### Application to e-Skins

The need to provide biomimetic artificial skins with sensitivity to changes rather than static stimulation has been recognised since the 1970’s^47,48^. Recent designs continue to attribute great importance to phasic sensing in robot proprioception^49^, tactile imaging^32^, prosthetics^50^, neurorobotics^51^, human-like sensing^52^, artificial discriminative touch^53^, or neuroprosthetics^54^, wearable Human–Machine interfaces^55^, inter alia.

## Methods

### Substrate

Polydimethylsiloxane (PDMS) (Sylgard 184, Dow corning, Midland, MI, USA) was mixed from a base and a curing agent in proportion of 10:1. After degassing, the gel was cast on cleaned silicon wafers coated with silicone releaser (Ease Release 200, Mann Release Technologies Inc., PA, USA). Spin coating removed excess gel. Samples were cured at ambient temperature for 24 h producing 0.6 mm membranes. Surfactant Triton X-100 (TX-1568-1, EM Science, Gibbstown, NJ, USA) (3 wt%) increased wettability^56^.

### Carbon nanotube solution

Aqueous solutions of commonly available purified Multi-Walled Carbon Nanotubes (MWCNT) (Cheap Tubes Inc., VT, USA), 0.5 vol% ${0.5}\;\hbox {mg mL}^{-1}$ suspended in deionised water with Sodium Dodecyl Sulfate (SDS) surfactant (${1}\;\hbox {mg mL}^{-1}$), were ultra-sonicated during 20 min receiving around  2000 J.

### Conductive polymer

Conductive polymer was PDMS based with Long-MWCNT carboxylic acid groups (Ad Nano Technologies LTD, Karnataka, India). Aqueous solutions were obtained by diluting PDMS and Long-MWCNT in $\hbox {CHCl}_3$. The solution was shear-mixed at 300 r.p.m., adding $\hbox {CHCl}_3$ to preserve viscosity. Curing agent was then added in proportion of 10:1.

### Sensor fabrication

**Figure 6:**
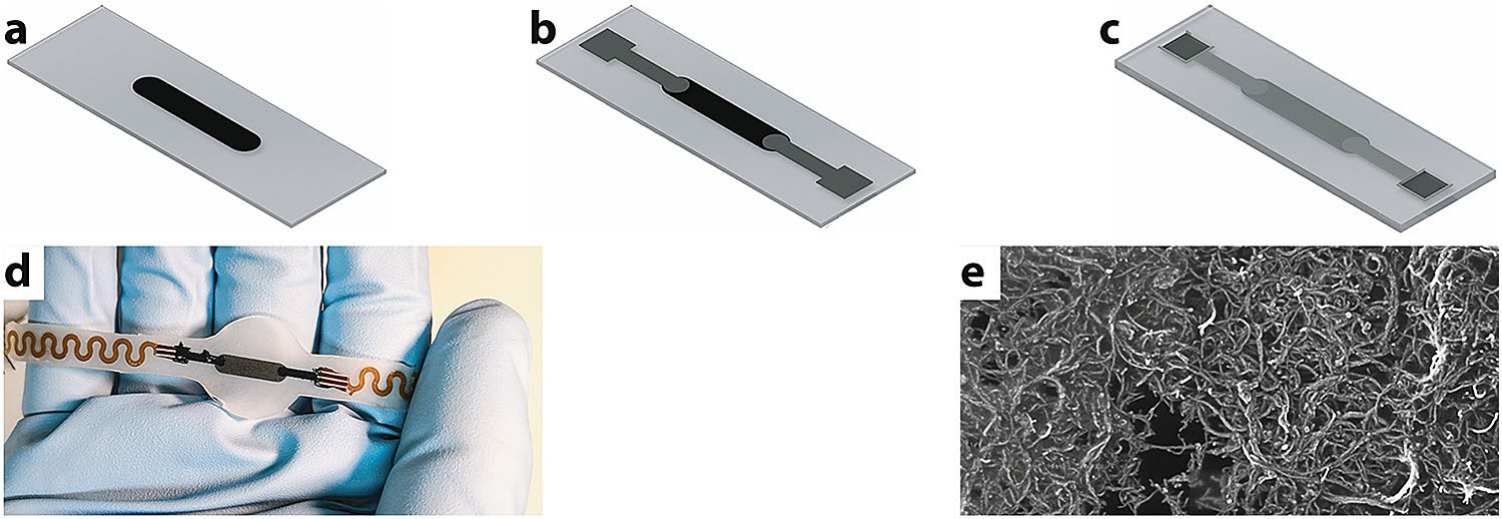
Fabrication. (**a**) PDMS plus Triton X, with deposited MWCNT film. (**b**) Conductive polymer patterned electrode. (**c**) Encapsulation. (**d**) Interconnection with printed circuits. (**e**) Scanning Electron Microscopy image of MWCNT film (Scan time ${50}\;{\upmu \hbox {s}}$, ${7.5}\;{\upmu \hbox {m}}$ Field-of-view).

MWCNT films were deposited by spray-coating aqueous solutions holding the substrate at ${130}\,^{\circ }\hbox {C}$ and airbrushing at 1.7 bar. Shadow masks were realised by laser cutting. Multiple coatings achieved desired resistivity. MWCNT films formed a ${1.5} \,\upmu \hbox {m}$ thick conductive network (Fig. 6a). Scanning Electron Microscopy revealed the conductive network (Fig. 6e). Conductive polymer stretchable interconnects were screen printed (Fig. 6b). Interconnects were cured at ${110}\,^{\circ }\hbox {C}$ to connect to flexible printed circuits and the sensors encapsulated in PDMS (Fig. 6c,d).

### Test bench and mechanical sequences

Resistance was measured with Keithley 2400 series SMU Instruments. In-house LabView program communicated with universal testing machine and Keithley SMU to command test sequences and log data. Compressive mechanical tests included deflections from 0.1 to 1.5 mm, loads from zero to 10 N and strain rates from 0.1 to ${5} {\text{mm}}\; {\text{s}}^{-1}$ at room temperature. Each trial comprised at least three cycles.

## Supplementary Information

Supplementary Information.

## Data Availability

The full set of data used to produce the results in this paper is available from the corresponding author upon request.
