# Supplementary material for: Artificial fast-adapting mechanoreceptor based on carbon nanotube percolating network
Source: Sci Rep. 2022 Mar 9;12:2818. doi: 10.1038/s41598-021-04483-2 (PMC8907247; doi:10.1038/s41598-021-04483-2)
Supplement: Supplementary file 1 — Supplementary Information. [file 41598_2021_4483_MOESM1_ESM.pdf]

Supplementary Information for

Artificial Fast-Adapting Mechanoreceptor Based on Carbon Nanotube Percolating Network

Cyril Bounakoff*, Vincent Hayward, Jonathan Genest, François Michaud, Jacques Beauvais
*Corresponding author. Email: cyril.bounakoff@usherbrooke.ca

Section S1: Generalized Maxwell Model
Section S2: Brief Summary of Piezoresistivity
Section S3: Percolation Networks

Supplementary Text

S1. Generalized Maxwell Model

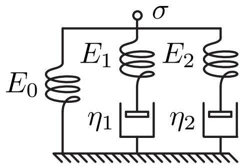

Figure S1. Maxwell model with three branches.

The generalised Maxwell model is commonly used to approximate the behaviour of polymers and tissues that exhibit viscoelastic proprieties. These materials exhibit a time-dependent response when stressed and strained. In this model, the standard elastic behaviour represented by the tensorial relationship, $\sigma = E_0\varepsilon$, where σ is the strain, ε is the stress, and E_0 is the quasi-static elastic modulus of the material, is augnented by a linear superposition of material models of the form,

$$\frac{\sigma}{\eta_i} + \frac{\dot{\sigma}}{E_i} = \dot{\varepsilon}\,,$$

where the η_i are viscosities. See in Fig. S1 for the case of $i = \{1,2\}$. The $\tau_i = \eta_i/E_i$ represent the different time constants of the material. Here, $\tau_1 = 0.2\,\mathrm{s}$ and $\tau_2 = 2.4\,\mathrm{s}$ where found to approximate well the viscoelastic matrix of the sensor cells.

S2. Standard Piezoresistive Gauge Equations

Consider a conductive cylinder of diameter D, or a slab of thickness D, of length L, of section S, of resistivity ρ, the resistance R is,

$$R = \rho\frac{L}{S} \quad \text{gives} \quad \frac{\Delta R}{R} \approx \frac{\Delta\rho}{\rho} + \frac{\Delta L}{L} - \frac{\Delta S}{S}\,.$$

Given ε_a and ε_t, the axial and transverse Cauchy strains, ε_a and ε_t , and ν the Poisson ratio, the dimensional changes are described by,

$$\varepsilon_\mathrm{a} = \frac{\mathrm{d}L}{L}, \quad \varepsilon_\mathrm{t} = \frac{\mathrm{d}D}{D}, \quad \frac{\mathrm{d}S}{S} = 2\frac{\mathrm{d}D}{D}, \quad \varepsilon_\mathrm{t} = -\nu\varepsilon_\mathrm{a}\,.$$

Thus, for small variations,

$$\frac{\mathrm{d}R}{R}(\varepsilon) = \frac{\mathrm{d}\rho}{\rho}(\varepsilon) + \varepsilon_\mathrm{a} - 2\varepsilon_\mathrm{a} = \frac{\mathrm{d}\rho}{\rho}(\varepsilon) + \varepsilon_\mathrm{a}(1+2\nu) = \frac{\mathrm{d}\rho}{\rho} - \varepsilon_\mathrm{t}\left(\frac{1}{\nu}+2\right).$$

In standard metal strain gauges it is said that in the variation of resistance as a function of strain, the term related the change in resistivity often dominates over the term related to dimensional changes. For certain materials the Poisson effect is small (viz. $\nu \approx 0.2$, in Si mono-crystals). For other materials such as polymers, $\nu \approx 0.5$, so for small variations,

$$\frac{\mathrm{d}R}{R}(\varepsilon) \approx \frac{\mathrm{d}\rho}{\rho}(\varepsilon) + 2\,\varepsilon_{\mathrm{a}} \approx \frac{\mathrm{d}\rho}{\rho}(\varepsilon) - 4\,\varepsilon_{\mathrm{t}}\,.$$

How $\mathrm{d}\rho/\rho$ varies according to ε depends on the choice of material.

Standard piezoresistivity seems unable to explain the behaviour of the sensing cell described in the main text since these relationships, even with non-uniform multi-axial strains under indentation, do not lend themselves to reversals in changes of resistance during steady increase or decrease of deflection. It would require the relationship from deflection to strain to be non-monotonic.

S3. Percolation Networks

In percolation networks, which are not continuous media but composites, resistivity is not distinguished from dimensional changes to model the change in resistance of a conducting element. The change in resistance is modelled in terms of distance changes between junctions owing to the heterogeneity of the medium.[1] The basic model is developed by writing the average resistance, R, of many junctions each of resistance, R_{j}, in series along many paths in parallel, and by neglecting the resistance, R_{c}, from junction to junctions,[2]

$$R = \frac{M(R_{\mathrm{j}} + R_{\mathrm{c}} \approx 0)}{N}\,,$$

where M is the number of junctions on a path and N is the number of paths. The tunnelling current, J, is a function of an electron's mass and charge, m and e, the potential and the potential barrier across the junction, V and ϕ, and s the length of the junction,

$$J = \frac{3\sqrt{2m\phi}}{2s}\left(\frac{2}{h}\right)^2 V\,\exp\left(-\frac{4\pi s}{h}\sqrt{2m\phi}\right)\,.$$

Given the effective cross-sectional area of all junctions, a^2, and regrouping some constants into $\gamma = 4\pi/(h\sqrt{2m\phi})$, and $\alpha = (8\pi h)/(3a^2\gamma e^2)$ we have,

$$R_{\mathrm{j}} = \frac{V}{a^2 J} = \alpha s\, e^{\gamma s}, \quad \text{thus} \quad R = \frac{M}{N}\alpha s\, e^{\gamma s}\,.$$

It follows that if under strain the length of the junctions change from s_0 to s, the resistance becomes,

$$\frac{R}{R_0} = \frac{s}{s_0} e^{\gamma(s-s_0)}\,.$$

In the case of uniform compressive strain strain, $\varepsilon_{\mathrm{a}} = (s - s_0)/s_0$, and $\varepsilon_{\mathrm{a}} = -2\varepsilon_{\mathrm{t}}$. Assuming that the junctions lengthen according to strain,

$$\frac{R}{R_0} = (1-\varepsilon)e^{\gamma s_0 \varepsilon_{\mathrm{a}}} \approx e^{\gamma s_0 \varepsilon_{\mathrm{a}}}, \quad \text{or} \quad \kappa = \frac{R - R_0}{R_0} = e^{\gamma s_0 \varepsilon_{\mathrm{a}}} - 1 = e^{-2\gamma s_0 \varepsilon_{\mathrm{t}}} - 1\,.$$

The relative variation decreases progressively slower as a function of uniform compressive strain which was empirically observed in composites loaded with nanotubes.[3,4] In the sensor cell described in the main text strain is like a Hertz-contact problem in the initial stages.

References

1. J. C. Dawson and C. J. Adkins. Conduction mechanisms in carbon-loaded composites. *Journal of Physics: Condensed Matter*, (43):8321, 1996.

2. X.-W. Zhang, Yi. Pan, Q. Zheng, and X.-S. Yi. Time dependence of piezoresistance for the conductor-filled polymer composites. *Journal of Polymer Science part B: polymer physics*, 38(21):2739–2749, 2000.

3. J. Genest, K. Su Kim, A. Sauvé, P. Boissy, G. Soucy, and J. Beauvais. Directly grown large area single-walled carbon nanotube films with very high sensitivity to normal pressure. *Journal of Applied Physics*, 111(2):023502, 2012.

4. J. H. Kang, C. Park, J. A Scholl, A. H. Brazin, N. M. Holloway, J. W. High, S. E. Lowther, and J. S. Harrison. Piezoresistive characteristics of single wall carbon nanotube/polyimide nanocomposites. *Journal of Polymer Science Part B: Polymer Physics*, 47(10):994–1003, 2009.
